# Pin1-Nanog expression in human glioma is correlated with advanced tumor progression

**DOI:** 10.3892/or.2013.2481

**Published:** 2013-05-23

**Authors:** YANG YANG, CHAO-SHI NIU, CHUAN-DONG CHENG

**Affiliations:** 1 Department of Neurosurgery, Anhui Provincial Hospital Affiliated to Anhui Medical University, Hefei, P.R. China; 2 Anhui Province Key Laboratory of Brain Function and Brain Disease, Hefei, P.R. China; 3 Anhui Provincial Stereotactic Neurosurgical Institute, Hefei, Anhui 230001, P.R. China

**Keywords:** glioma, Pin1, Nanog, expression

## Abstract

The stemness gene Nanog has been shown to play an important role in tumor development, including glioma. Nanog is phosphorylated at multiple Ser/Thr-Pro motifs, which promotes the interaction between Nanog and the prolyl isomerase Pin1, leading to Nanog stabilization by suppressing its ubiquitination. The present study investigated the expression and relationship of Pin1 and Nanog in human gliomas. Significantly higher mRNA and protein expression levels of Pin1 and Nanog were demonstrated in 120 glioma specimens of different pathological grades by RT-PCR, immunohistochemistry staining and western blot analysis. The relative levels of Pin1 expression, as well as Nanog expression, were significantly positively correlated with pathological grade. Moreover, a positive correlation of Pin1 and Nanog expression in human gliomas was noted. Co-localization of Pin1 and Nanog was observed in the perinuclear space in the cytoplasm of glioma cells detected by immunofluorescence staining. Significantly positive correlation between Pin1 and Nanog in gliomas indicated that Pin1 and Nanog may be related to tumorigenesis and development of glioma cells.

## Introduction

The most common malignant primary brain tumors are gliomas. Despite aggressive surgery, radiation and chemotherapy, the median survival is only 12–15 months for glioblastoma multiforme (GBM) (1). It is critical to explore the mechanism involved in the development and progression of glioma and to find new therapeutic targets. Few biomarkers have thus far been integrated into clinical practice.

Nanog is a stem cell transcription factor that is essential for embryonic development, reprogramming normal adult cells and malignant transformation and progression (2). Oncogenesis has long been considered an abnormal embryogenesis and tumor cells share a few biological properties with ESCs (3). Several tumor cell types have previously been reported to express Nanog (4,5). Downregulation of Nanog by histone deacetylase inhibitor apicidin could lead to cell cycle arrest, differentiation and apoptosis in human embryonic carcinoma NCCIT cells (6). Our previous research demonstrated the overexpression of Nanog in glioma tissues and brain tumor stem cells (BTSCs) compared with normal brain tissues, indicating that Nanog may contribute to the existence of BTSCs and may be related to tumorigenesis of the cerebrum by maintaining the undifferentiated state of glioma cells (7).

Phosphorylation on serine or threonine residue preceding proline (Ser/Thr-Pro) is a major intracellular signaling mechanism. The conformation of certain phosphorylated Ser/Thr-Pro bonds is regulated specifically by the prolyl isomerase Pinl. Pin1 is the only one of the prolyl isomerase family that can recognize the phosphorylated Ser/Thr-Pro motif (pS/pT-P motif) and induce the cis/trans conversion of the proline bond (8,9). It has been reported that Pin1 is markedly overexpressed in several types of human cancer (10–12). Pinl might amplify and translate multiple oncogene signal mechanisms during oncogenesis and function as a pivotal catalyst for multiple oncogenic pathways.

Nanog is phosphorylated at several Ser/Thr-Pro motifs, which promotes the interaction between Nanog and the prolyl isomerase Pin1 (13). The interaction is important for Nanog stabilization by suppressing its ubiquitin dependent degradation. Disruption of Pin1-Nanog interaction in ESCs suppresses their capability to self-renew and to form teratomas in immunodeficient mice (13). In human colorectal cancer, it has been found that both Pin1 and Nanog are located in the perinuclear space in the cytoplasm where they may interact to affect cell proliferation and maintain the stemness of human colorectal cancer (2).

In the present study, we first investigated the expressions of Pin1 and Nanog in gliomas, as well as the correlation between them. For both Pin1 and Nanog, their mRNA and protein expressions were detected and found highly expressed in human gliomas and positively correlated with pathological grade of patients with gliomas. Furthermore, we frequently observed a positive relationship between Pin1 and Nanog in gliomas. We also confirmed that the co-location of Pin1 and Nanog was mainly in the perinuclear space in the cytoplasm of glioma cells. However, further study is required to determine the precise role of the Pin1-Nanog pathway, and the mechanism of Pin1-Nanog transcriptional regulation in gliomas.

## Materials and methods

### Clinical sample collection

The patients had received no treatment prior to the craniotomy. Human glioma tissues (n=120) were obtained from patients with newly diagnosed glioma who had received no therapy before sample collection and had undergone resection at the Anhui Provincial Hospital Affiliated to Anhui Medical University between 2007 and 2010. Normal brain specimens were acquired from 7 trauma patients for whom partial resection of normal brain tissue was required. All specimens were collected in the operating room immediately (≤15 min) after tumor resection and were then snap frozen in liquid nitrogen and stored at −80°C. The enrollment criteria for the glioma patients in the present study were: glioma diagnosis by pathology based on World Health Organization (WHO) grading; no prior antiglioma treatment; suitable formalin fixed, paraffin-embedded tissues and frozen tissues were available. All glioma samples were verified by pathological analysis and classified according to the WHO 2007 classification standard. There were 22 low-grade (WHO grade II) and 98 high-grade tumors (WHO grades III 42 and IV 56). None of the patients had received chemotherapy, immunotherapy and radiotherapy prior to specimen collection. Ethics approval for human subjects was obtained from the Research Ethics Committee of the Anhui Provincial Hospital Affiliated to Anhui Medical University and informed consent was obtained from each patient.

### RT-PCR

Reverse transcription-polymerase chain reaction (RT-PCR) was performed as previously described (7). Total RNA was extracted from the human glioma samples with TRIzol reagent (Invitrogen, Carlsbad, CA, USA) and treated with DNase (Fermentas, Vilnius, Lithuania) to remove DNA contamination. RNA (200 ng to 1 μg) and M-MLV (Takara, Shiga, Japan) and oligo-dT (Takara) were used for cDNA synthesis. PCR was performed with 2X Taq Plus PCR Master Mix (Tiangin, China). The primer sequences and the size of the amplified product were: Pin1 (427 bp), 5′-TCAGGCC GAGTGTACTAC-3′ (forward) and 5′-CGGAGGATGAT GTGGATG-3′ (reverse); Nanog (403 bp), 5′-ATGCCTGT GATTTGTGGGCC-3′ (forward) and 5′-GCCAGTTGTTT TTCTGCCAC-3′ (reverse); β-actin (252 bp), 5′-ATGGATGA TGATATCGCCGCGCTC-3′ (forward), and 5′-TTTCTCCAT GTCGTCCCAGTTGG-3′ (reverse).

β-actin was used as the internal control. In semi-quantitative RT-PCR, standardized template amounts were used to amplify cDNA for 30–35 cycles. The PCR products were separated on 1.5% agarose gels by electrophoresis. The intensity of the bands was determined using the Quantity One software (Bio-Rad Laboratories).

### Western blotting

Western blotting was performed as previously described (7). Membranes were probed with mouse anti-human Nanog polyclonal antibody (1:100; Abcam) or rabbit anti-human Pin1 polyclonal antibody (1:500; Abcam) at 4°C overnight or mouse monoclonal anti-β-actin antibody (1:1,000 dilution) (Beyotime Institute of Biotechnology, Nanjing, China) for 1 h at room temperature followed by the horseradish peroxidase (HRP)-conjugated goat anti-mouse or goat anti-rabbit IgG antibody (ZSGB-BIO, Beijing, China). Immunoblots were visualized by chemiluminescence using an ECL Detection system (BeyoECL Plus; Beyotime Institute of Biotechnology). The intensity of the bands was determined using the Image-Pro Plus 6.0 software.

### Immunohistochemical analysis

Immunohistochemical analysis was performed as previously described (7,14). Slides were deparaffinized and rehydrated following standard methods. A microwave antigen retrieval procedure was carried out for 20 min in citrate buffer (pH 6.0). Hydrogen peroxide was used to block non-specific peroxidase reaction. Sections were blocked with normal goat serum (20 min), then incubated with rabbit anti-human Pin1 polyclonal antibody (1:200; Abcam, Cambridge, UK) or mouse anti-human monoclonal antibody Nanog (1:100; Abcam) for 12 h at 4°C followed by treatment with biotinylated secondary antibody; color reactions were performed with diaminobenzidine (DAB) (Sigma). The sections were lightly counterstained with hematoxylin. Negative control sections were incubated with PBS instead of the primary antibody.

In the present study, positive cells were scored based on nucleus and cytoplasm staining of Nanog protein. The number of positive immunostained cells out of 100 in 10 random high-power fields (Olympus BX51; Tokyo, Japan) was scored (7,15). Nanog expression was classified semi-quantitatively according to the following criteria: 0 when <5% of glioma cells discretely expressed Nanog in their nucleus and cytoplasm; 1+ when >5 to <25% of glioma cells discretely expressed Nanog in their nucleus and cytoplasm; 2+ when >25% to <50% of tumor cells are immunopositive; 3+ when >50% of morphologically unequivocal neoplastic cells discretely expressed Nanog in the nucleus and cytoplasm. We considered samples scored as 2+ and 3+ as high expression, while 0 and 1+ as low expression. The Pin1 expression was evaluated visually and semi-quantified according to previous studies (16–18). The scoring system was based on both the intensity and the labeling frequency percentage. Cases with Pin1 score 0–2 were assigned to the low Pin1 expression group, and cases with Pin1 score 3–6 to the high Pin1 expression group. Sections were scored by two independent pathologists with no knowledge of the associated clinical data. In cases of occasional scoring discrepancy, consensus was always achieved after a discussion of the findings.

### Immunofluorescence staining

The U87 human glioma cell line used in the present study was purchased from the Chinese Academy of Sciences Type Culture Collection. The cells were routinely maintained in high glucose DMEM supplemented with 10% FBS, 100 U/ml penicillin, and 100 mg/ml streptomycin at 37°C in a humidified incubator with a 5% CO_2_ atmosphere.

Immunofluorescence staining studies were performed as previously described (7,18). U87 cells were grown on coverslips for 24–48 h. They were then fixed with 4% paraformaldehyde for 20 min at room temperature and washed three times with 0.2% Triton X-100/PBS for 15 min for permeabilization. The coverslips were blocked with 10% normal goat serum for 30 min and then incubated at 4°C overnight with primary antibody Nanog (1:100 dilution) or Pin1 (1:200 dilution), followed by FITC-or TRITC-conjugated secondary antibodies. The cells were counterstained with 4′,6-diamidino-2-phenylindole (DAPI) (Sigma, St. Louis, MO, USA). The images were acquired using an Olympus BX51 fluorescence microscope.

### Statistical analysis

All statistical analyses were performed by SPSS 17.0 software package for Windows. Data in the text and figures are expressed as the means ± SD. The independent Student's t-test or one-way analysis of variance (ANOVA) was used to compare the expression level of Pin1 or Nanog between groups. Correlation analysis of the expression levels of Pin1 and Nanog was performed using the Spearman rank-sum test. P<0.05 was considered to indicate a statistically significant difference in all tests.

## Results

### Pin1 is highly expressed in human gliomas and is positively correlated with pathological grade

**Figure 1:**
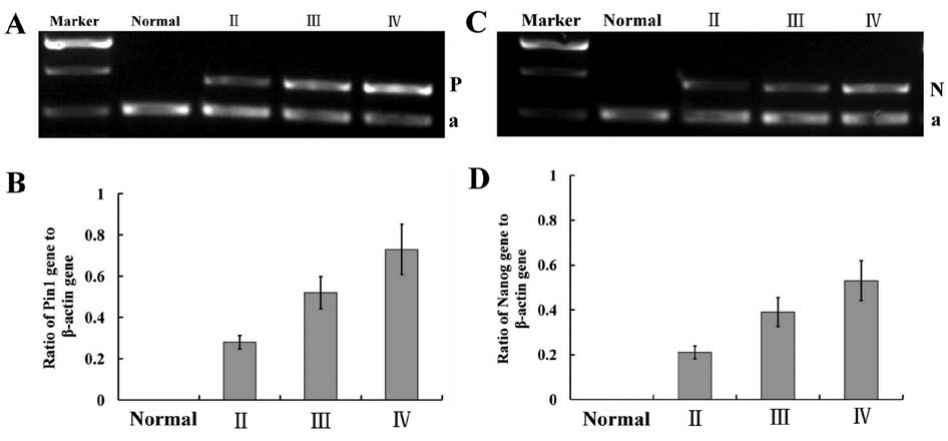
Expression of Pin1 and Nanog gene in differential pathological grade glioma tissues. (A) Expression of Pin1 mRNA by RT-PCR in different pathological grade glioma tissues (normal brain tissues as control). (B) Histogram representing relative level of Pin1 mRNA by RT-PCR (F=21.814, P<0.01, ANOVA). (C) Expression of Nanog mRNA by RT-PCR in different pathological grade glioma tissues (normal brain tissues as control). (D) Histogram representing relative level of Pin1 mRNA by RT-PCR (F=18.381, P<0.01, ANOVA). P, Pin1 (427 bp); N, Nanog (403 bp); a, β-actin (252 bp).

**Figure 2:**
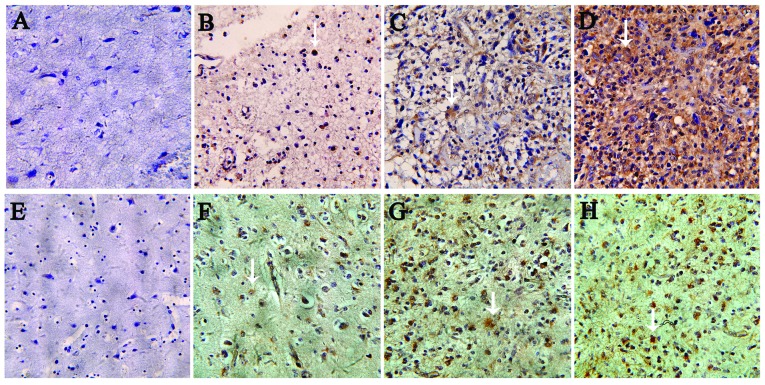
Immunohistochemical analysis of the expression patterns of Pin1 and Nanog in differential pathological grade glioma tissues. (A–D) Pin1 immunohistochemical staining of paraffin sections of gliomas (magnification, ×400). (E–H) Nanog immunohistochemical staining of paraffin sections of gliomas (magnification, ×400). (B) Expression of Pin1 in WHO grade II tissue; Pin1 expression was primarily localized in the nuclei of tumor cells (white arrow). (C) Expression of Pin1 in WHO grade III tissue; Pin1 expression was localized in both the cytoplasm and nuclei of glioma cells (white arrow). (D) Expression of Pin1 in WHO grade IV tissue; Pin1 was expressed in both the cytoplasm and nuclei of glioma cells (white arrow). (F–H) Expression of Nanog in WHO grade II, III, IV glioma tissues; Nanog showed mainly nuclear or perinuclear staining with some cytoplasmic localization (white arrow). (A and E) Normal brain tissues.

**Figure 3:**
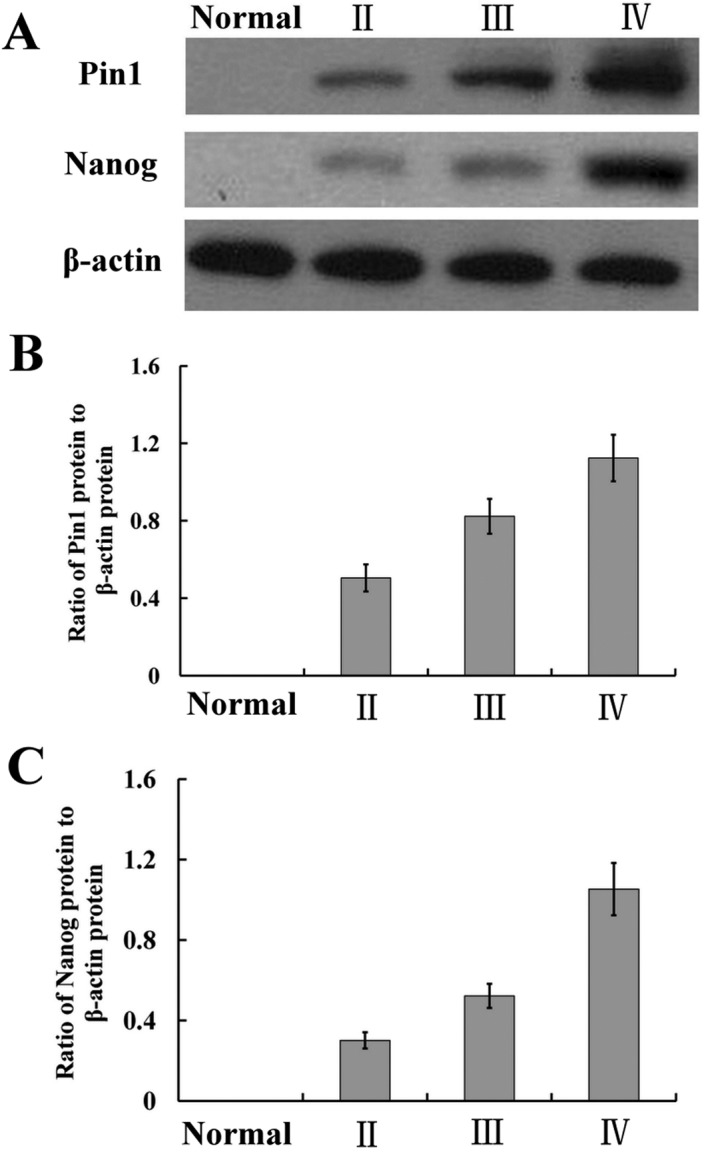
Expression of Pin1 and Nanog protein in differential pathological grade glioma tissues. (A) The expressions of Pin1 and Nanog protein by western blotting in different pathological grade glioma tissues (normal brain tissues as control). (B) Histogram representing the relative level of Pin1 protein as determined by western blot analysis (F=22.962, P<0.01, ANOVA). (C) Histogram representing the relative level of Nanog protein as determined by western blot analysis (F=42.691, P<0.01, ANOVA).

We initially analyzed the expression profiles of 120 gliomas to examine whether Pin1 was enriched in glioma tissues. We used the primers described above to investigate Pin1 mRNA expression levels in the glioma tissues of different pathological grade (Fig. 1). Densitometric evaluation of the relative expression showed that the mRNA level of Pin1 in the high-grade primary gliomas was significantly higher than that in the low-grade gliomas (F=21.814, P<0.01) (Fig. 1). When observed by H&E staining and excluding necrotic and hemorrhagic tissues, the glioma cells within gliomas were relatively homogeneous. The immunohistochemical staining results showed that 95 (79.17%) glioma samples were positively stained and 25 (20.83%) glioma samples were negatively stained. Among the normal brain specimens, all 7 (100%) specimens were negatively stained. In addition, Pin1 expression was mainly confined to the nuclei in low grade glioma in a lower degree of enrichment and weak expression, but exhibited enhanced expression in both the cytoplasm and nuclei of high grade glioma. Moreover, a marked positive correlation was noted between the expression of Pin1 and pathological grade (r=0.279, P<0.01) (Fig. 2 and Table I). By contrast, no evident expression was observed in the 7 normal brain samples. Following the above observations, we carried out western blot analysis to confirm the relationship between the Pin1 expression and pathological grade. As expected, a similar differential expression pattern was observed; the higher expression of Pin1 correlated with a more highly malignant glioma (F=22.962, P<0.01) (Fig. 3).

### Nanog is highly expressed in human gliomas and is positively correlated with pathological grade

Our previous research demonstrated the overexpression of Nanog in glioma tissues and BTSCs compared with normal brain tissues (7). Our current data also reveal that Nanog showed predominantly nuclear or perinuclear staining with some cytoplasmic localization in glioma cells. The immunohistochemical staining results showed that 88 (73.33%) glioma specimens were positively stained and 32 (26.67%) glioma specimens were negatively stained. The normal brain tissues were all negatively stained. Nanog mRNA and protein expressions were highly expressed in gliomas, particularly WHO IV glioma samples (F=18.381, P<0.01, ANOVA; F=42.691, P<0.01, ANOVA). Moreover, the protein expression levels of Nanog were positively correlated with pathological grade (r=0.211, P<0.05) (Figs. 1–3 and Table I).

### Correlation between Pin1 and Nanog expression in human gliomas

Positive immunostaining of Pin1 and Nanog was observed in glioma cells. Based on the hierarchical scores of the immunohistochemical staining described above, we proceeded to analyze the correlation between Pin1 and Nanog in gliomas. The results indicated that the expression levels of Pin1 and Nanog were positively correlated in gliomas (r=0.209, P<0.05) (Table II), which suggest a high correlation between the levels of Pin1 and Nanog in glioma development.

### Subcellular localization and coexpression of Pin1 and Nanog in glioma cells

**Figure 4:**
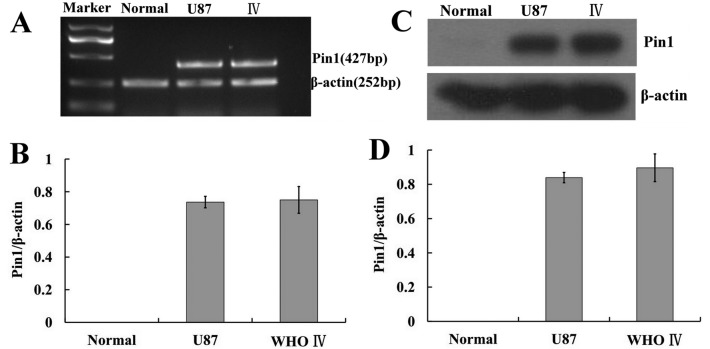
Expression of Pin1 in U87 glioma cells. (A) Expression of Pin1 mRNA as determined by RT-PCR in U87 glioma cells and WHO IV glioma tissues. (B) Histogram representing the relative level of Pin1 mRNA (P>0.05, independent Student's t-test). (C) Western blot analysis of U87 cells and WHO IV glioma tissues. (D) Histogram representing the relative level of Pin1 protein as determined by western blot analysis (P>0.05, independent Student's t-test).

**Figure 5:**
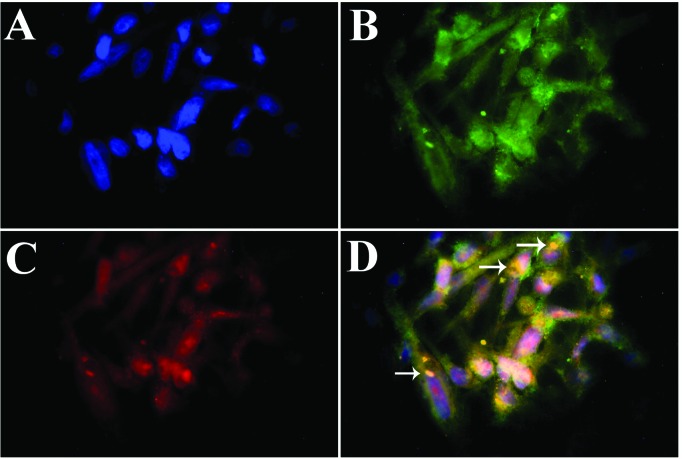
Detection of the co-expression of Pin1 and Nanog in U87 glioma cells by immunofluorescence staining. (A) nuclei (DAPI). (B) Pin1 (FITC); Pin1 was expressed in both the cytoplasm and the nuclei of glioma cells. (C) Nanog (TRITC); Nanog showed mainly nuclear or perinuclear staining with some cytoplasmic localization. (D) Merged view; Pin1 and Nanog were co-located in the perinuclear space in the cytoplasm of glioma cells (white arrow). Magnification, ×1,000.

In previous research, Nanog mRNA and protein expression in U87 glioma cells was confirmed (7). In the present study, Pin1 mRNA and protein expression in U87 cells was examined using RT-PCR and western blotting, respectively (Fig. 4). RT-PCR analysis of cells revealed the expected 427-bp Pin1 band in both the U87 cells and WHO IV glioma tissues (t=0.259, P>0.05). No obvious band was observed in the normal brain tissues. Western blot analysis confirmed that Pin1 was highly expressed in both U87 cells and WHO IV glioma tissues (t=1.138, P>0.05). For further analysis, immunofluorescence staining was performed to detect the subcellular localization and coexpression of Pin1 and Nanog. Pin1 was expressed in both the cytoplasm and nuclei of U87 glioma cells. At the same time, Nanog showed mainly nuclear and perinuclear staining with some cytoplasmic localization. The majority of glioma cells coexpressed Pin1 and Nanog (Fig. 5B and C). Furthermore, Pin1 and Nanog were co-located in the perinuclear space in the cytoplasm of glioma cells (Fig. 5D), where they may interact and have a cytoplasmic function to affect glioma cells.

## Discussion

Gliomas are the most common primary tumors in the central nervous system (CNS). Malignant gliomas are the most lethal tumors originating in the CNS, which account for 70% of gliomas with a high recurrence and mortality rate (1). The most biologically aggressive subtype of gliomas is glioblastoma multiforme (GBM), a tumor associated with a rather poor prognosis. Although major advances have been made in surgery, chemotherapy and radiotherapy for gliomas, the life expectancy of patients with GBM and anaplastic astrocytoma (WHO grade III) remains short, with a median survival of approximately only 14–16 months and 2–5 years, respectively (19). Advances in the treatment of malignant gliomas require improved understanding of the biology and the molecular mechanisms of glioma development and progression, as well as the elucidation of novel molecular markers and signaling pathways. Identification of the sets of genes that are differentially expressed in different grade glioma specimens and normal brain tissues is important to understand the molecular basis of glioma, to predict patient prognosis and to develop novel therapeutic strategies.

Pin1, which catalyzes cis-to-trans conformational switches of target proteins presenting the phospho Ser/Thr-Pro (pS/T-Pro) motif, has received considerable attention as a cofactor that regulates the phosphorylation of several target proteins (20). Previous studies showed that Pin1 is overexpressed in a number of common tumors (10–12), and several of its target proteins have an altered phosphorylation profile (20,21). Such Pin1 activity is correlated with a change in target protein stability through a ubiquitin-mediated mechanism (22–24). Pin1 dependent conformational changes are a unique signaling mechanism essential in regulating numerous cellular functions. For instance, functional inactivation of RUNX3, a tumor suppressor, is frequently observed in various types of cancer, including glioma and breast cancer (25–27). Expression of Pin1 inversely correlates with the expression of RUNX3 in human breast cancer samples. Of note, Pin1 recognizes four phosphorylated Ser/Thr-Pro motifs in RUNX3 via its WW domain. Binding of Pin1 to RUNX3 could suppress the transcriptional activity of RUNX3 by inducing the ubiquitination and proteasomal degradation of RUNX3 (25). Similarly, Fbw7, a well-characterized major tumor suppressor, is the substrate recognition component of the Skp1-Cullin-F-box (SCF)-type E3 ligase complex, which is frequently inactivated by mutation or genetic deletion in various types of human cancer (28–30). Min *et al*(31) found that Pin1 directly interacts with Fbw7 to disrupt Fbw7 dimerization. As a result, Pin1 blocks the ability of Fbw7 to mediate substrate degradation, but promotes Fbw7 self-ubiqutination instead. These results support the idea that Pin1 promotes the progression of cancer (32). In our research, we found significantly higher Pin1 mRNA and protein expression in glioma samples as compared with the normal brain tissue samples. An association between higher Pin1 expression and aggressive grades of gliomas was also demonstrated, which suggests that Pin1 may participate in the pathogenesis of gliomas, which are defined as poorly differentiated according to purely histopathological criteria (7). However, whether these findings are associated with Pin1 dependent conformational changes, which change target protein stability through a ubiquitin-mediated mechanism, remains to be further analyzed.

Nanog, a core transcription factor reported by Mitsui *et al*(33), plays a critical role in maintaining self-renewal and pluripotency of ESCs by regulating cell fate of pluripotent inner cell mass (ICM) (34–36). Apart from controlling such ‘stemness’ properties, the role of Nanog in tumorigenesis has attracted significant attention. Increasing evidence suggests that most tumors are heterogeneous. Of these, a small subset of cells, known as cancer stem cells, arise from mutated adult stem/progenitor cells possessing stem cell-like properties, which are responsible for tumor growth, metastasis, chemoresistance and, thus, cancer recurrence (37). Only by targeting these populations of cells, which share several key biological properties with normal stem cells, can the disease be cured (38,39). Nanog overexpression has already been detected in a number of human tumors, including glioma cells, and is involved in some oncogenic pathways, suggesting that Nanog plays a critical role in tumor genesis and progression (2,4–7,37,40). Moretto-Zita *et al*(13) reported that Pin1 could induce conformational change in Nanog by isomerizing the pS/T-Pro bonds, leading to the inhibition of ubiquitin-proteasome-dependent degradation of Nanog. The disruption of the interaction between Pin1 and Nanog suppressed ESC self-renewal. Pin1 plays critical roles in various types of cancer by changing target protein stability through a ubiquitin-mediated mechanism. However, why Pin1 facilitates ubiquitin-mediated degradation of tumor suppressors but also stabilizes Nanog, a novel oncogene, by suppressing its ubiquitination has yet to be fully clarified and requires further research.

In the present study, the association between Pin1 and Nanog in human gliomas, the subcellular localization and coexpression of Pin1 and Nanog in glioma cells were investigated. We have shown that high Pin1 and Nanog expressions were detected in glioma specimens by RT-PCR, western blotting and immunohistochemical analysis. We also confirmed that Pin1 was expressed in both the cytoplasm and nuclei of glioma cells, which was consistent with Ryo *et al*(12) who reported that Pin1 expression was found to be confined to the nuclei in low grade astrocytoma at relatively low expression levels but exhibited enhanced expression in both the cytoplasm and nuclei of anaplastic astrocytoma and glioblastoma. Furthermore, Nanog showed mainly nuclear and perinuclear staining with some cytoplasmic localization in glioma cells. This finding was consistent with previous results which found the Nanog protein was located in both the nuclei and in the cytoplasm of breast carcinoma, prostate cancer and glioma cells (4,41,42). We further confirmed that majority of glioma cells coexpressed Pin1 and Nanog, which were co-located in the perinuclear space in the cytoplasm of glioma cells, where they may interact and have a cytoplasmic function to affect glioma cells. Additionally, Pin1 and Nanog expression were positively correlated in glioma tissues, indicating they may interact to affect cell proliferation and maintain the cell viability and stemness of glioma. On the basis of these findings, we hypothesize that the Pin1-Nanog pathway may be important in the tumorigenesis of the gliomas. Targeting the Pin1-Nanog pathway may be an approach to improve the therapeutic intervention for poorly differentiated gliomas.

In conclusion, we have shown that Pin1 and Nanog expression in human gliomas appears to be associated with the pathogenesis of gliomas. Furthermore, Pin1 expression is positively correlated and co-located with Nanog expression in glioma. Pin1 and Nanog may play an important role in glioma tumorigenesis through interaction. Further research is required to elucidate the difference in response and control of expression of Pin1 and Nanog in glioma, and to explore if Pin1 could act as a ubiquitination switch in regulating glioma cellular functions through Nanog conformational changes.

## Figures and Tables

**Table I:** Positive correlation between Pin1-Nanog expression and pathological grade in human glioma tissues.

		Pin1 expression			Nanog expression		
					
WHO	Cases	Low	High	P-value	Low	High	P-value
II	22	13	9		17	5	
III	42	20	22	0.002	20	22	0.020
IV	56	14	42		24	32	

**Table II:** Correlation between Pin1 and Nanog expression in human glioma tissues.

	Low Pin1	High Pin1	P-value
Low Nanog	30	31	0.022
High Nanog	17	42	
